# An SIR-type epidemiological model that integrates social distancing as a dynamic law based on point prevalence and socio-behavioral factors

**DOI:** 10.1038/s41598-021-89492-x

**Published:** 2021-05-13

**Authors:** Maritza Cabrera, Fernando Córdova-Lepe, Juan Pablo Gutiérrez-Jara, Katia Vogt-Geisse

**Affiliations:** 1 Centro de Investigación de Estudios Avanzados del Maule (CIEAM), 3480112 Talca, Chile; 2 Facultad de Ciencias Básicas, Universidad Católica del Maule, 3480112 Talca, Chile; 3 Vicerrectoria de Investigación y Postgrado, Universidad Católica del Maule, 3480112 Talca, Chile; 4 Facultad de Ingeniería y Ciencias, Universidad Adolfo Ibáñez, 7941169 Santiago, Chile

**Keywords:** Applied mathematics, Epidemiology

## Abstract

Modeling human behavior within mathematical models of infectious diseases is a key component to understand and control disease spread. We present a mathematical compartmental model of Susceptible–Infectious–Removed to compare the infected curves given by four different functional forms describing the transmission rate. These depend on the distance that individuals keep on average to others in their daily lives. We assume that this distance varies according to the balance between two opposite thrives: the self-protecting reaction of individuals upon the presence of disease to increase social distancing and their necessity to return to a culturally dependent natural social distance that occurs in the absence of disease. We present simulations to compare results for different society types on point prevalence, the peak size of a first epidemic outbreak and the time of occurrence of that peak, for four different transmission rate functional forms and parameters of interest related to distancing behavior, such as: the reaction velocity of a society to change social distance during an epidemic. We observe the vulnerability to disease spread of close contact societies, and also show that certain social distancing behavior may provoke a small peak of a first epidemic outbreak, but at the expense of it occurring early after the epidemic onset, observing differences in this regard between society types. We also discuss the appearance of temporal oscillations of the four different transmission rates, their differences, and how this oscillatory behavior is impacted through social distancing; breaking the unimodality of the actives-curve produced by the classical SIR-model.

## Introduction

Epidemics and pandemics are a thread for public health. More pandemic situations such as the current pandemic caused by the viral disease COVID-19 may come in the future. Such a pandemic can cause a devastating public health, social and economic impact across the world. In a pandemic situation governments may be forced to impose and promote restrictive measures to control disease spread. The approach different societies take may vary according to cultural, political and economic realities of each country^1^. Restrictive measures may eventually have to be relaxed due to the economic and social impact that these can provoke, especially in poorer societies^2^, while finding a balance between health and economic factors, and trusting on companies and individuals to implement and maintain protective measures^3^. Social distancing is one of the main recommended individual protective measures during pandemic situations caused by directly transmitted diseases^4^. Social distancing has been shown to be an effective measure for controlling disease burden for instance during the SARS epidemic of 2003 in Hong Kong^1^, or during the current COVID-19 pandemic^5, 6^. Epidemic situations force individuals to develop a change in their social behavior. For instance, there is evidence that the appearance of new behaviors could be conditioned by fears, worries and anxiety among individuals, which recently has been measured by the use of *The Fear of COVID-19 Scale*^7^. Hence, in such situations, societies are forced to make cultural changes that strengthen the awareness for public health. These cultural changes if maintained, could help prevent the dissemination of infectious diseases and future epidemics^8^.

We focus on studying through a mathematical model the epidemiological effects of keeping a certain social distance when encounters are not to be avoided during an epidemic outbreak of a directly transmitted disease. Mathematical modeling of human behavior is an essential tool to guide control strategies, impulsed for instance to prevent infection in risk groups^8, 9^. To incorporate in mathematical epidemiological models variables or parameters that describe social behavior is an important challenge^10^. With our model, we seek to: first, compare disease dynamics for different types of societies under different distance dependent transmission rate functional forms; second, understand and describe how the dynamics of the social distance—depending on the observed point prevalence—affects the transmission rates and disease dynamics; third, identify epidemiological and social/cultural factors relevant for disease mitigation.

## Methodological aspects

There exists an extensive number of mathematical models that explain, characterize and project the evolution of different infectious diseases that affect humans^11–15^. In addition to present a compartmental model that classically describes disease dynamics, we incorporate social distancing as a dependent variable following a dynamic law based on point prevalence and socio-behavioral factors. Theories of human behavior state that there exist environmental factors (e.g., climate, demographic growth, location) and psycho-social aspects (e.g., degree of aggregation, economic prosperity, culture) that influence the distance that individuals maintain from each other in their daily lives^8, 16–18^. Additionally, human groups define cultural norms that can be classified into the following types^19^: (i) Contact cultures, which relate through close personal distance emphasizing physical contact; (ii) non-contact cultures, in which individuals keep further distance from each other, avoiding physical contact. For instance, contact cultures are found in Southern Europe, Latin America and the Arab countries, while non-contact cultures are found in North America, North of Europe and Asia.

When modeling a disease, it would be best to have a clear understanding about how interactions between people occur, for then recognize social patterns. There are social studies that provide information on social distancing, in particular on the average distance between susceptible and infectious individuals, which is very useful to understand disease spread^20^. There are methods based on statistics that determine the distance distribution using the number of infectious events associated to all possible susceptible-infectious cases^20^. Other studies state that the probability of infection between susceptible and infectious decreases with distance according to a formulation of the Power Law^21^. In general, we assume that an average behavior– connected to social and cultural characteristics of a population– offers, up to a certain level useful information to answer questions at population level, in terms of ecological and epidemiological nature^22^. There are also studies in the literature related to social distancing, which incorporate the effect on disease dynamics of frequency-duration of physical contact and distances that exist between households in social settings^23, 24^. In the aforementioned study^24^, the recorded data are social distances of 1,821 individuals living in Southern China, aggregated in different environments, such as: age and rural or urban conditions. As a result, the study reveals that distance is inversely proportional to the probability of infection. In addition, it was shown that social contacts and their duration decrease with chronological age. Those results provide contact patterns that are consistent with similar research studies conducted in European countries^19^. For technical simplicity and lack of more accurate information, in this study we assume a uniformly distributed distancing behavior, in populations aggregated by culture. In other words, we assume that all individuals with the same cultural background follow the same social distancing behavior; as we describe in the next section.

There exist several articles that study the spread of infectious diseases related to human behavior through mathematical models. One of the first generalizations of the Kermack-McKendrick deterministic epidemic model^25^ in that respect was given by Capasso and Serio in 1978^26^, where they present the force of infection in an SIR (Susceptible–Infected–Removed) compartmental type model of differential equations as a function *g*(*I*), which saturates for large levels of infectives in the population, changing the transmission rate of the classical SIR model from constant to non-constant. After a study that these authors conducted about the cholera epidemic in Bari, Italy, they wanted to reflect—with the saturation of the force of infection—the psychological effect in the population that leads to adopt more self-protective measures when the number of infected individuals is high. Also, in^27^, a non-linear force of infection including a saturation function represents the influence of human behavioral change in a cholera model due to health education, hygiene and sanitation practices. In^28^, an SIR model with exponential saturation of the force of infection of the form $\beta (I)=\mu (1-e^{-aI})$ is presented, with the intent to capture disease dynamics as an outbreak progresses and behaviors change, where for instance the parameter *a* is reduced by mask wearing. There are several other articles incorporating similar non-linear force of infection terms (see^28–33^ and references therein). Additionally, the article by D’Onofrio et al.^34^ incorporates a non-constant transmission rate $\beta (M)$ that depends not only on the current number of infectives but also on *M*, representing an information index that summarizes the current and past history of disease prevalence. Their results show that social behavioral change may trigger oscillations in the infectious population. On the other hand, Pedro et al.^35^ extend an SIR type model incorporating the effect of social support for school and workplace closure on disease dynamics, and study socio-economic conditions for a second COVID-19 wave. In the aforementioned study, the authors define a transmission rate that captures the impact of closure through a function of time. This function is governed by a dynamic law explained by Imitation Dynamics^36^ to describe population-level support for closure. The article in^9^ also uses Imitation Dynamics to present the competing dynamics between a resident pathogenic strain and a mutant strain with higher virulence. That article studies a population in which individuals learn and develop a behavior to protect each other. Other dynamic mathematical models have included behavior by dividing the population into different risk groups, and this way studying the epidemiological effects based on social distancing while including individuals’ risk perception, awareness, fear, cooperation or activity level^37–46^. Specifically, a model that quantifies the epidemiological impact of the size of groups of individuals who do or do not follow responsible behavior can be seen in^43^. The study shows how the Basic Reproduction Number (an epidemiological threshold that generally determines disease dynamics^47^) and disease prevalence changes according to each responsible individual. It also discusses the necessity of quantifying the effect that distance between individuals has on disease transmission. On the other hand, the article in^40^ studies media induced social distancing in an SIR type model including an extra social distancing compartment, whose influx rate is influenced by media; and the authors in^39^ present a compartmental model that stratifies the population not only by disease status but also by disease awareness status.

Also, there exists evidence for the changing temporal behavior of disease transmission in epidemic or pandemic settings^28, 48–55^, which justifies extending an SIR type model by incorporating a non-constant transmission rate. In particular, there are articles—some as a result of the high demand in understanding COVID-19—that fit mathematical models in order to represent the decrease in the transmission rate^27, 28, 31, 49^. For instance, the article in^49^ includes a time varying exponential decay log function for the transmission rate, to capture this way the early decreasing shape of the transmission rate of COVID-19 thought to be due to enforced lockdowns and disease mitigation interventions.

We consider a deterministic mathematical model based on ordinary differential equations that divide the human population into Susceptible–Infectious–Removed (SIR)^56^, and extend it including a non-constant transmission rate. The transmission rate of a disease depends on the effective contact rate of individuals, which depends on individual distancing behavior, and determines the occurrence of infection^10^. The novelty of the model we present is to assume that the transmission rate is represented by different functional forms that depend inversely on a dynamic distance that individuals keep from each other. The dynamics of this distance depends on the point prevalence of the disease and the resistance to change, which comes from the necessity people feel to return to their natural social distance. We make two assumptions regarding the average distance between individuals: (a) in the absence of disease, individuals tend to maintain a certain average distance from each other, which we will call *natural-distance*, and (b) in the presence of disease, individuals respond by increasing their social distance according to the appreciation of point prevalence levels, and hence the *natural-distance* becomes a dynamic distance that we will call *interaction-distance*. We first compare epidemic curves, and the size and timing of the first appearing epidemic peak, for society types that differ according to social distancing behavior related to assumptions (a) and (b). We study the disease dynamics of these societies for different transmission rate functional forms that are *interaction-distance* dependent, and for different parameter values appearing in these transmission rates. Then, we discuss the temporal dynamics of the four transmission rate functions, how their shape is explained through social distancing behavior and their added practical significance to the classical SIR model when modeling the propagation of infectious diseases.

## Cultural distance as risk factor for disease transmission

In the field of semiotic, the discipline that studies the organization of space in terms of linguistic communication is called Proxemic^18^. In the present work, we will take few elements of this area, in particular related to the types of space that surround the human body—their limits and use–, which could help us characterize distancing behavior in different cultural settings, essential for disease transmission.

Generally speaking, a person defines his or her distance range or degree of physical contact according to the social interconnection she/he experiences with the counterpart (e.g., family, friends, colleagues or strange). Some studies also point out that personal differences such as: personality, age, gender, social conditions, etc., are crucial for a person to decide his or her personal distance boundaries^8, 17, 18, 57^. Nevertheless, the main factor that determines the distance that individuals keep from each other is cultural related^18^, which is associated with the geographical region the population is located. As mentioned before, we consider an average distancing behavior assumed equal for all individuals within the same cultural background. Thereby, different average distancing behaviors might affect differently the transmission rate of the disease, leading to cultural changes in disease dynamics.

The term Proxemic is conceptualized by the notion of personal space when referred to the form by which human beings physically interact with each other, either with peers or objects^18^. In this respect, physical distance is correlated with the social closeness that individuals keep from each other, being characterized in the following way: (i) intimate, (ii) personal, (iii) social and public. Specifically, to each social relationship type corresponds a personal space, which is configured by concentric bubbles, of radius: (i) from 0 to 0.45 (m) for intimate distance ; (ii) from 0.45 to 1.2 (m) for personal distance ; (iii) more than 3.5 (m) for social distance. Latin communities for instance tend to interact socially keeping less distance compared to Anglo-American societies. Indeed, the work in^58^, titled *Proxemics and Tactility in Latin America* states that there exist different ways of proxemic communication between individuals belonging to different Latin American countries and even between gender encounters (man-man; man-woman; or woman-woman). The aforementioned study revealed that the encounter between gender, together with the country of origin are determinant factors that affect the average distance individuals keep from each other. It was performed through a multivariate analysis of variance to determine if gender and culture have an effect on human-distances with pairs using groups of individuals from Costa Rica, Panama and Colombia respectively. As a result, it was proven that Costa Rica interacts significantly closer than the rest of the countries located on the south and the mean distance for female pairs is significantly smaller than other gender's encounters.

A global study in the field of Cross Cultural Psychology revealed a comparative interpersonal distancing world wide, using a large data set of 8,943 participants from 42 countries^19^. According to those authors, Southern European, Latin American and Arabian countries are considered closer cultures with notable physical contact behaviors; whilst North America, Northern Europe and Asian countries prefer more distant encounters and non contact behaviors. As a result, a list of global comparative social distances^19^, comprised by countries with small, medium and large social distancing allowed among peers is shown in Table 1. It shows the average natural social distance given by the culture of each country. In the Americas, the frequency of physical contacts and their distances decrease progressively as we move from North America to South America. Therefore, it is impossible to determine a common universal contact index for all cultures^18^. We include this cultural difference using a specific base parameter that is interpreted as the distance that individuals would keep to each other culturally in the absence of the disease. This is the parameter we refer to as *natural-distance*.

**Table 1:** Average social *natural-distances measured in meters (m)* for different countries of origin.

Type of social *natural-distance*	Distance interval (m)	Country of origin
Small	[0; 1)	Italy–Argentina–Bulgaria–Greece
		Ukraine–Russia–Slovakia–Austria
		Serbia–Peru–Spain
Medium	[1; 1.2]	USA–Germany–Indonesia–Estonia
		England–Poland–Canada–Norway
		China–Brazil–Nigeria–South Korea
		India–Switzerland–Kenya–Portugal
		Czech Republic–Malaysia–Iran
		Pakistan–Croatia–Mexico–Ghana
		Hong Kong
Large	$(1.2 ;\infty )$	Uganda–Hungary–Saudi Arabia
		Romania–Turkey

(Information was obtained from the article by Sorokowska et al.^19^).

## Distance-contagion model

We consider a Susceptible–Infectious–Removed (SIR) type model with recovery and transmission rates given respectively by $\gamma$ and $\beta (D)$. The latter, is assumed to be dependent on the average dynamic distance that individuals usually keep from each other, which we denote *D* and call *interaction-distance*. The functional form that $\beta (D)$ takes will be introduced in the next section. We assume in our model, that the dynamic for *D* depends on the level of infectious and that in the absence of disease the *interaction -distance* returns to its natural equilibrium $D_{*}$, which represents the *natural-distance* of the society. We also suppose no demographic change, no immigration, and a constant total population size $N=S+I+R$. The system of differential equations that determines the dynamic is:

1 $$\begin{aligned} \left\{ \begin{array}{ccl} S'&{}=&{}-\beta (D)\,S\,(I/N) \\ I'&{}=&{}+\beta (D)\,S\,(I/N)-\gamma \, I\\ R'&{}=&{}+\gamma \, I\\ D'&{}=&{}-\lambda _{1}\,(D-D_{*})+\lambda _{2}\,(I/N), \end{array}\right. \end{aligned}$$

with positive initial conditions $S(0)=S_0$, $I(0)=I_0$, $R(0)=R_0$, $D(0)=D_0$. The rate $\lambda _{1}\ge 0$ [1/*time*] measures the rate of resistance, per distant unit, to change distancing behavior. It measures how fast individuals return to their *natural-distance* $D_*$, or in other words, the rate at which individuals return to natural distance habits, given by their culture. $\lambda _{2}\ge 0$ [*distance*/*time*] determines the reaction-velocity by which change occurs according to how people perceive point prevalence levels. Observe that if $\lambda _1=0$ there is no resistance and *D* increases ($D'>0$) proportional to point prevalence: *D* increases steeply if people react fast (large $\lambda _2$) and increases slightly if they react slow (small $\lambda _2$); on the contrary, if $\lambda _1>>0$ individuals tend to return to their *natural-distance* $D_*$ fast, so there is a large resistance to change their natural way of living. Also, if $\lambda _2=0$, the population does not react to point prevalence levels and hence the distance decreases and tends to the equilibrium $D_{*}$, as long as $D_0>D_{*}$; if on the contrary $\lambda _2>>0$, the population is very perceptive and reacts quickly to change, even when point prevalence levels may be low.

Observe that, when solving the last equation from the system in Eq. (1), we obtain

2 $$\begin{aligned} D(t)=D_{*}+(D_{0}-D_{*})e^{-\lambda _{1}t}+\frac{\lambda _{2}}{N} \int _{0}^{t} I(s)e^{-\lambda _{1}(t-s)} \, ds, \end{aligned}$$

which is a function such that if $D_0=D_*$ (i.e. society follows its natural distancing behavior when the first infectious person appears), then $0 \le D(t) - D_{*} < \lambda _{2}/\lambda _{1}$.

Moreover, its asymptotic behavior ($t\rightarrow \infty$) is as follows

3 $$\begin{aligned} \lim _{t\rightarrow \infty } D(t)=D_{*}+\frac{\lambda _{2}}{N} \displaystyle \lim _{t \rightarrow \infty } {\mathscr {J}}(t),\quad \text{ with }\quad {\mathscr {J}}(t) =\int _{0}^{t} I(s)e^{-\lambda _{1}(t-s)} \, ds. \end{aligned}$$

Since in Eq. (1), the $S\rightarrow I \rightarrow R$ flow is uni-directional, we have $I(+\infty )= 0$. Hence, given $\epsilon >0$, there exists $\tau >0$ such that $0\le I(s)<\varepsilon$ for $s>\tau$. Therefore, ${\mathscr {J}}(t)$ in Eq. (3) is bounded by ${\mathscr {J}}(\tau )+\varepsilon [1-e^{-\lambda _{1} (t-\tau )}]/\lambda _{1}$ and by making $t \rightarrow \infty$ we conclude that $D(+\infty )=D_{*}$ exponentially, see Fig. 6b, such that the *interaction-distance* converges to the constant *natural-distance* of the society. Notice that once expressing *I*/*N* in terms of *D*, we obtain for *S* that $S'/S=-\beta (D)\{ D'+\lambda _{1}(D-D_{*})\}/\lambda _{2}$, which when integrating over [0, *t*] provides the following expression

4 $$\begin{aligned} \ln \left( \frac{S_{0}}{S(t)}\right) =\frac{1}{\lambda _{2}} \int _{D_{*}}^{D(t)}\beta (u)\,du +\frac{\lambda _{1}}{\lambda _{2}} \int _{0}^{t}\beta (D(u))\,\{D(u)-D_{*}\}du. \end{aligned}$$

We denote $S_{\infty }$ the value of *S* for infinite time, and obtain

5 $$\begin{aligned} S_{\infty }=S_{0}\,/\exp \left\{ \frac{\lambda _{1}}{\lambda _{2}} \int _{0}^{\infty }\beta (\Delta D(u)+D_{*})\,\Delta D(u)\,du\right\} , \end{aligned}$$

where $\Delta D:=D-D_{*}$. As is to be expected, the epidemics will end with more susceptibles if: the reaction velocity ($\lambda _2$) is large, the resistance ($\lambda _1$) and/or the *natural-distance* $(D_*)$ is small. Additionally, if $\beta (\cdot )$ is given by Eq. (8) (shown in the next section), with $\nu =1$, we have that $S_{\infty }=S_{0}\,/\exp \left\{ (\lambda _{1}/\lambda _{2}) \beta _{*}D_{*}L(\infty ) \right\}$, with $L(\tau )=\tau -D_{*}\int _{[0,\tau ]}\{1/D(u)\} du$, an increasing function.

Many factors determine change in behavior, and in particular the dynamics of the *interaction-distance* between individuals. It may be difficult to quantify parameters related to those changes, such as the rate of resistance to change ($\lambda _1$), or the reaction-velocity to change ($\lambda _2$). But, assumptions could be made on how on average the population thinks. In general, there are different types of behavioral changes, as described in^16^, such as: definite or momentary; local or global; uni-causal or multi-causal; group influenced or individual; superficial or profound. When a change in habits occurs, it is generally difficult to maintain over time and according to the authors in^16^, maintaining it depends on cultural re-education initiatives.

## Distance dependent transmission rate and the basic reproduction number (${\mathscr {R}}_0$)

In this section, we will describe how the transmission rate $\beta (\cdot )$ varies with social distance *D*. We assume a base line transmission rate $\beta _{*}>0$ and a scaling distance $\bar{D}_*$, such that $\beta (\bar{D}_{*})=\beta _{*}$. In what follows, we will present four functional forms.

6 $$\begin{aligned} \beta _{1}(D)=\beta _{*}\left[ \frac{2\bar{D}_{*}}{\bar{D}_{*}+D}\right] ^{\nu }, \quad \nu >0. \end{aligned}$$

$\beta _1(D)$, was inspired on $\beta _2(D)$ below, and found in^59^. On one hand, it similarly decreases in a convex form, but keeping its own structural geometry and qualitative differences.

7 $$\begin{aligned} \beta _{2}(D)=\beta _{*}\left[ \frac{2\bar{D}_{*}^{\nu }}{\bar{D}_{*}^{\nu }+D^{\nu }}\right] , \quad \nu >0. \end{aligned}$$

$\beta _2(D)$, was introduced in^59^ using a Maximum Likelihood estimation for the Blue Tongue virus serotype 8 epidemic with a data set from Netherlands and Germany and $\beta _3(D)$ described in Eqn. (8) was obtained through a parameter estimation of the transmission rate with data from Belgium based on distances between farms. In both cases *D*, represents the inter farm distance; $\beta _{*}$ the initial rate of transmission or base line transmission, and $\bar{D}_{*}$ a scaling distance^59^. Meanwhile $\nu$ is a parameter that measures the decrease in the infectious rate with distance at farm level^60^.

8 $$\begin{aligned} \beta _{3}(D)&=\beta _{*}\left[ \frac{\bar{D}_{*}}{D}\right] ^{\nu }, \quad \nu >0. \end{aligned}$$

9 $$\begin{aligned} \beta _{4}(D)&=\beta _{*}\exp \left[ 1-\left( \frac{D}{\bar{D}_{*}}\right) ^{\nu }\right] , \quad \nu >0. \end{aligned}$$

The form for $\beta _4(D)$ was introduced in^57^ in the context of social mixing patterns in rural and urban areas of Southern China aimed to quantify the human interactions targeted for better understanding on the transmission of respiratory infectious diseases. In this study, the contact duration was assigned as an integer number multiplied by the number of individuals following an exponential distribution to each contact event.

Notice that the functional forms given in Eqs. (6)–(9), are decreasing, convex functions such that $\displaystyle \lim _{D\rightarrow \infty } \beta _i(D)=0$ for $i\in \{1,2,3,4\}$; but for small values of *D* they differ in the following way: $\beta _{1}(0^{+})=2^{\nu }\beta _{*}$, $\beta _{2}(0^{+})=2\beta _{*}$, $\beta _{4}(0^{+})=e \beta _{*}$ and $\beta _{3}(0^{+})=\infty$.

In what follows of this article, we are going to consider that the scaling distance $\bar{D}_*$ is an average of the distance types D_* from Table 1 and hence is fixed at $\bar{D}_*=1.05$. This way, we can observe: for societies that are experiencing medium *interaction-distance* $D=1.05$, the transmission rate is the base line transmission rate $\beta _*$; for societies with *interaction-distance* $D<1.05$ the transmission rate is larger than $\beta _*$; and, for societies with *interaction-distance* $D>1.05$, the transmission rate is smaller than $\beta _*$.

**Figure 1:**
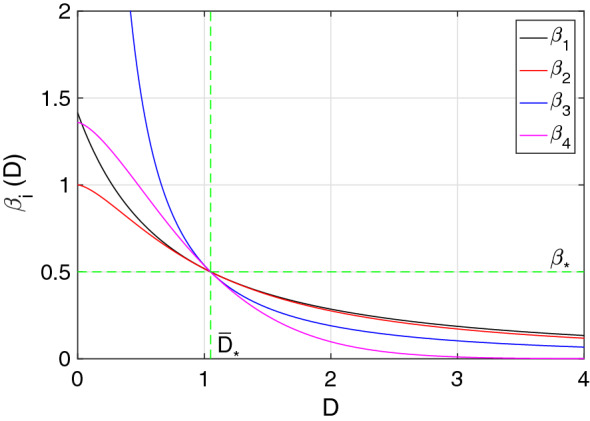
The different transmission rate functional forms $\beta _i(D)$, $i=1,2,3,4$ are pictured. The scaling distance was taken to be $\bar{D}_{*}=1.05$ for all transmission rates, which was considered to be an average of the distance types D_* from Table 1. The other parameters were chosen to be $\beta _{*}=0.5$ and $\nu =1.5$.

Figure 1 shows the transmission curves of the four functional forms from Eqs. (6)–(9): $\beta _{1}(D)$ (black), $\beta _{2}(D)$ (red), $\beta _{3}(D)$ (blue) and $\beta _{4}(D)$ (purple), with scaling distance $\bar{D}_*=1.05$. It can be seen that for $D=\bar{D}_*=1.05$, $\beta _i(\bar{D}_*)=\beta _{*}$ for $i=\{1,2,3,4\}$, which is the base line constant transmission rate. This means that once the *interaction-distance* *D* is close to $\bar{D}_{*}=1.05$, all transmission rates are similar and close to $\beta _{*}$. On the contrary, if the *interaction-distance* reaches the type small, medium or large (see Table 1), the transmission rates differ from each other accordingly. Observe that before $\bar{D}_{*}=1.05$, the order of the transmission rates is $\beta _{2}(D)<\beta _{1}(D)<\beta _{4}(D)<\beta _{3}(D)$ and afterwards it changes to $\beta _{4}(D)<\beta _{3}(D)<\beta _{2}(D)<\beta _{1}(D)$.

**Figure 2:**
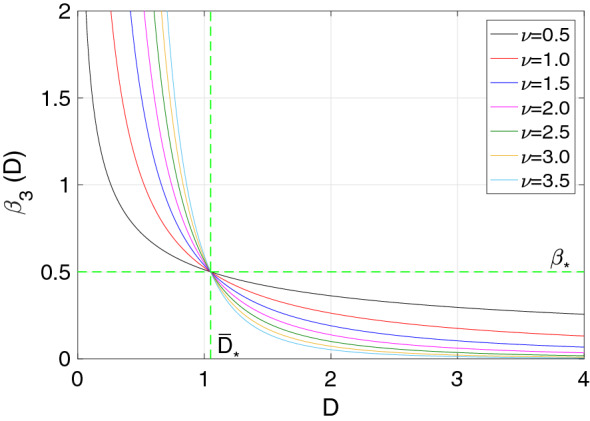
The transmission rate functional form for $\beta _3(D)$ is pictured. The scaling distance was taken to be $\bar{D}_{*}=1.05$ for all transmission rates, which was considered to be an average of the distance types D_* from Table 1. The base line transmission rate was chosen to be $\beta _{*}=0.5$.

Figure 2 shows $\beta _3(D)$ for the scaling distance $\bar{D}_*=1.05$ and for different values of $\nu$. From the picture it can be appreciated that for societies experiencing an *interaction-distance* *D* less than $\bar{D}_*=1.05$, the transmission rate increases with increasing $\nu$, and, on the contrary, that for societies with *interaction-distance* *D* greater than $\bar{D}_*=1.05$, the transmission rate decreases with increasing $\nu$.

The basic reproduction number, ${\mathscr {R}}_0$, is an important threshold quantity that generally determines the course of an epidemic and the corresponding dynamics of the system describing it, such that usually an epidemic peak occurs if ${\mathscr {R}}_0>1$, and on the other hand, the disease is not able to invade the population if ${\mathscr {R}}_0<1$^47^. Linearizing the system in Eq. (1) around the disease free state and considering D_0=D_*, $\beta _i(\cdot )$ reduces to $\beta _i(D_*)$, $i=1,2,3,4$, and using a similar approach as in^61^ we obtain the known form for ${\mathscr {R}}_0$ for an SIR model without demography—whose value depends on the *natural-distance* $D_*$ for each society type from Table 1–, which is

10 $$\begin{aligned} {\mathscr {R}}_{0}=\frac{\beta _i(D_*)}{\gamma }, \qquad i=1,2,3,4. \end{aligned}$$

## Numerical results

We present numerical simulations to study disease dynamics for different societies under distinct transmission rate functional forms and distance-related parameters. We also show the practical significance of these four functional forms according to their dynamics in time and dependency on *interaction-distance*. The software MATLAB^62^ was used to create all figures in this section, as well as Figs. 1 and 2 from the previous section.

### Disease transmission and dynamics under different *natural-distance* ($D_*$) assumptions

**Figure 3:**
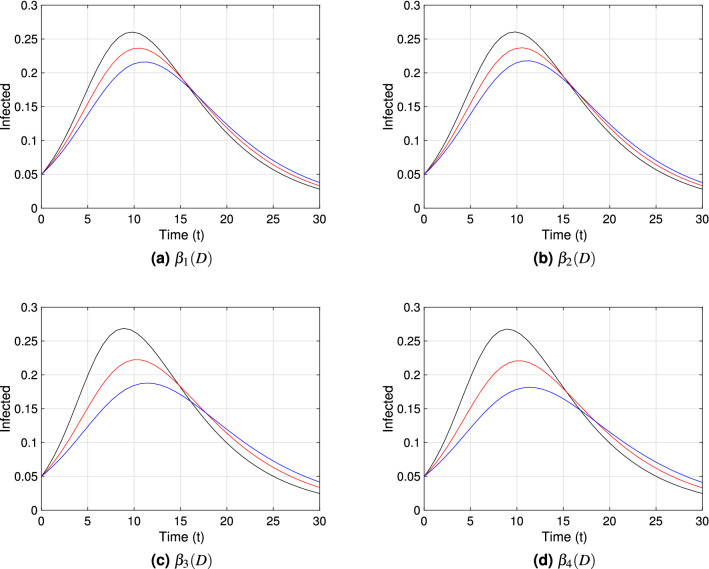
Point prevalence, *I*(*t*)/*N*, from the system in Eq. (1) with respect to time for each transmission functional form and $\nu =0.5$. Black, red and blue curves correspond to $D_*=0.75\;({\rm m})$, $D_*=1.05\;({\rm m})$, and $D_*=1.35\;({\rm m})$ respectively. These correspond to societies of small ([0, 100)), medium ([100, 120]) and large ($[120,\infty )$) *natural-distance* types respectively (see Table 1). The other parameter values are fixed at $\beta _{*}=0.5$; $\gamma =0.2$, $\nu =0.5$, $\lambda _{1}=0.03$, $\lambda _{2}=0.3$. The ${\mathscr {R}}_0$ value for each society are for (**a**) and (**b**): ${\mathscr {R}}_0=2.7$ (black), ${\mathscr {R}}_0=2.5$ (red), ${\mathscr {R}}_0=2.3$ (blue); (**c**) and (**d**): ${\mathscr {R}}_0=3.0$ (black), ${\mathscr {R}}_0=2.5$ (red), ${\mathscr {R}}_0=2.2$ (blue). The initial condition $D(0)=D_*$ was used for each type of society.

**Figure 4:**
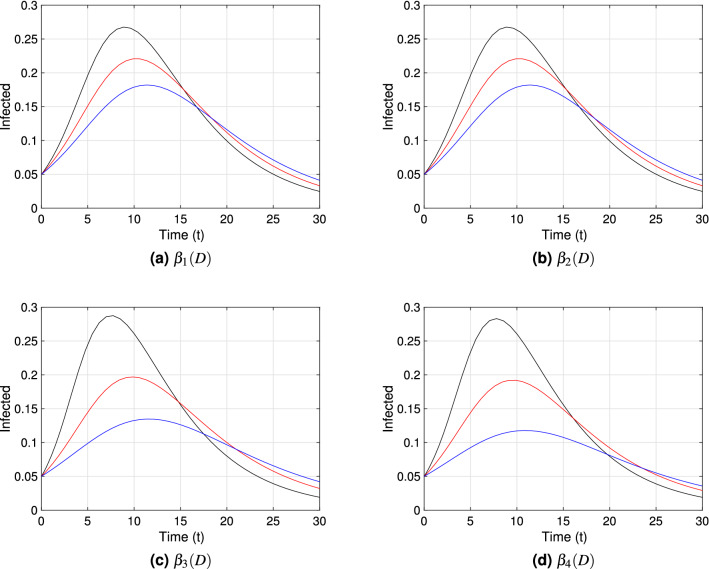
Point prevalence, *I*(*t*)/*N*, from the system in Eq. (1) with respect to time for each transmission functional form and $\nu =1$. Black, red and blue curves correspond to $D_*=0.75\;({\rm m})$, $D_*=1.05\;({\rm m})$, and $D_*=1.35\;({\rm m})$ respectively. These correspond to societies of small ([0, 100)), medium ([100, 120]) and large ($[120,\infty )$) *natural-distance* types respectively (see Table 1). The other parameter values were taken to be $\beta _{*}=0.5$; $\gamma =0.2$, $\nu =1$, $\lambda _{1}=0.03$, $\lambda _{2}=0.3$. The ${\mathscr {R}}_0$ value for each society are for (**a**) and (**b**): ${\mathscr {R}}_0=2.9$ (black), ${\mathscr {R}}_0=2.5$ (red), ${\mathscr {R}}_0=2.2$ (blue); (**c**): ${\mathscr {R}}_0=3.5$ (black), ${\mathscr {R}}_0=2.5$ (red), ${\mathscr {R}}_0=1.9$ (blue); (**d**): ${\mathscr {R}}_0=3.3$ (black), ${\mathscr {R}}_0=2.5$ (red), ${\mathscr {R}}_0=1.9$ (blue). The initial condition $D(0)=D_*$ was used for each type of society.

**Figure 5:**
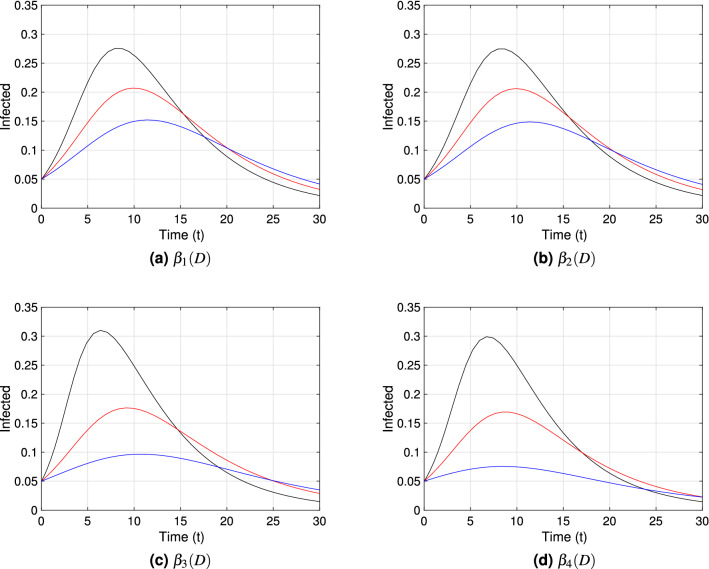
Point prevalence, *I*(*t*)/*N*, from the system in Eq. (1) with respect to time for different transmission functional forms and $\nu =1.5$. Black, red and blue curves correspond to $D_*=0.75\;({\rm m})$, $D_*=1.05\;({\rm m})$, and $D_*=1.35\;({\rm m})$ respectively. These correspond to societies of small ([0, 100)), medium ([100, 120]) and large ($[120,\infty )$) *natural-distance* types respectively (see Table 1). The other parameter values were taken to be $\beta _{*}=0.5$; $\gamma =0.2$, $\nu =1.5$, $\lambda _{1}=0.03$, $\lambda _{2}=0.3$. The ${\mathscr {R}}_0$ value for each society are for (**a**) and (**b**): ${\mathscr {R}}_0=3.1$ (black), ${\mathscr {R}}_0=2.5$ (red), ${\mathscr {R}}_0=2.0$ (blue); (**c**): ${\mathscr {R}}_0=4.1$ (black), ${\mathscr {R}}_0=2.5$ (red), ${\mathscr {R}}_0=1.7$ (blue); (**d**): ${\mathscr {R}}_0=3.7$ (black), ${\mathscr {R}}_0=2.5$ (red), ${\mathscr {R}}_0=1.5$ (blue). The initial condition $D(0)=D_*$ was used for each type of society.

Each graph within Figs. 3, 4 and 5 shows the point prevalence curve (*I*(*t*)/*N*) from the system in Eq. (1) with respect to time, for the different transmission rate forms as in Eqs. (6)–(9): $\beta _{1}(D)$, $\beta _{2}(D)$, $\beta _{3}(D)$ and $\beta _{4}(D)$, with scaling distance $\bar{D}_*=1.05$. The colors correspond to the *natural-distances* $D_{*}$ value in the system given in Eq. (1), for three different types of societies: (i) small social *natural-distance* (black, $D_*=0.75$), within the range [0, 1) [*m*]; (ii) medium social *natural-distance* (red, $D_*=1.05$), within the range [1, 1.2] [*m*]; and (iii) large social *natural-distance* (blue, $D_*=1.35$), within the range $(1.2,\infty ) ({\rm m})$. We consider $\nu =0.5$, $\nu =1$ and $\nu =1.5$ in Figs. 3, 4 and 5 respectively, to account for the effect of the shape of the transmission functions on disease dynamics. To study the first impact of an epidemic, the time frame chosen for the mentioned figures shows the peak of a first epidemic outbreak, considering that our model may allow for further smaller peaks (see Fig. 9 in the next subsection).

Comparing Figs. 3, 4 and 5, we can observe especially for $\beta _3(D)$ and societies of small *natural-distance* type (black) that, the higher $\nu$ is the larger is the size of the peak and the sooner does the peak occur (compare subfigure (c) in Figs. 3, 4, 5). Also, significant differences in point prevalence levels can be observed between societies of different *natural-distance* types (small, medium or large), especially for $\beta _3(D)$ and large $\nu$ values (see Figs. 4c, 5c). Figures 4c and 5c show clearly that societies of small *natural-distance* type (black) show the greatest increase in peak size but also the largest shift in the occurrence of the peak when compared to others. In general, the smaller the *natural-distance* type of the society is, the sooner does the peak occur. These culturally driven differences are less if we observe the dynamics for $\beta _1(D)$ and $\beta _2(D)$, especially for small $\nu$ values, and are most noticeable for $\beta _3(D)$ and $\beta _4(D)$ for large $\nu$ values.

**Figure 6:**
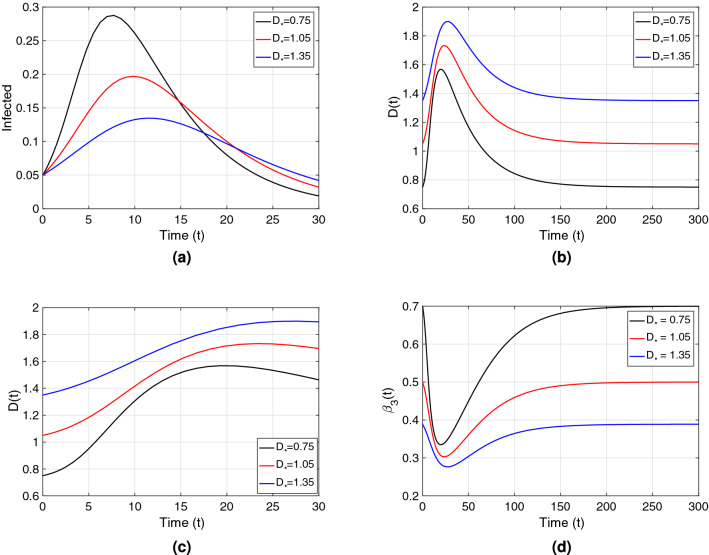
(**a**) Point prevalence, *I*(*t*)/*N*, from the system in Eq. (1) with respect to time. (**b**) Distance *D*(*t*) kept by individuals through the epidemic. (**c**) Zoomed version of (**b**). (**d**) Transmission rate $\beta _3(t)$ $=\beta _3(D(t))$ from Eq. (8). All plots in the figure consider $\beta _{3}(D)$ as the transmission rate. Black, red and blue curves correspond to $D_*=0.75\;({\rm m})$, $D_*=1.05\;({\rm m})$, and $D_*=1.35\;({\rm m})$ respectively. These correspond to societies of small ([0, 100)), medium ([100, 120]) and large ($[120,\infty )$) *natural-distance* types respectively (see Table 1). The other parameter values were taken to be $\beta _{*}=0.5$; $\gamma =0.2$, $\nu =1$, $\lambda _{1}=0.03$, $\lambda _{2}=0.3$. The ${\mathscr {R}}_0$ value for each society type is: ${\mathscr {R}}_0=3.5$ (black), ${\mathscr {R}}_0=2.5$ (red), ${\mathscr {R}}_0=1.9$ (blue). The initial condition $D(0)=D_*$ was used for each type of society.

For each type of society, Fig. 6a describes the dynamics of infected individuals (*I*(*t*)/*N*); Fig. 6b,c shows the evolution of the *interaction-distance* *D*(*t*) from the system in Eq. (1), kept by individuals though the course of the epidemic for a certain distance-related parameter set; and Fig. 6d describes the corresponding temporal dynamics of the transmission rate $\beta _3(t)$ $=\beta _3(D(t))$ from Eq. (8). The *interaction-distance* *D*(*t*) reaches a peak, which occurs after the epidemic peak (compare Fig. 6a,c). After attaining the peak, the *interaction-distance* curves converge to their respective culturally determined *natural-distance* $D_*$ (see Fig. 6b). Figure 6a shows that the peak of the infected curve, as discussed earlier, shifts according to the *natural-distance* type of the society (determined by the value of $D_*$), as do the peaks of the distance curves (see Fig. 6b), in reaction to the disease peak. One can also observe from Fig. 6b that the absolute change in *interaction-distance* is largest for societies of small *natural-distance* type (black curve), compared to other types. The transmission rate $\beta _3(t)$ behaves as expected, inversely proportional to *interaction-distance*, being the societies of large *natural-distance* types the ones with the smallest transmission rate as well as the smallest absolute change in transmission (see Fig. 6d).

**Figure 7:**
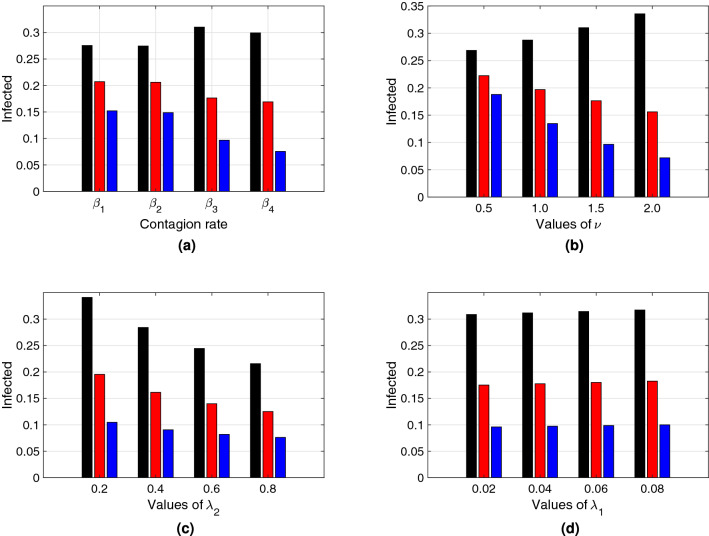
Size of the peak of a first epidemic outbreak from the system in Eq. (1) with respect to (**a**) contagion rates $\beta _i$, $i=1,2,3,4$, (**b**) measure of decrease of transmission rate with distance $\nu$, (**c**) reaction velocity to change $\lambda _2$, (**d**) rate of resistance to change $\lambda _1$, for different society types: Black, red and blue bars correspond to $D_*=0.75\;({\rm m})$, $D_*=1.05\;({\rm m})$, and $D_*=1.35\;({\rm m})$ respectively. These correspond to societies of small ([0, 100)), medium ([100, 120]) and large ($[120,\infty )$) *natural-distance* types respectively (see Table 1). The height of each bar represents the size of the peak of the epidemic curve. The other parameter values were taken to be $\beta _{*}=0.5$ and $\gamma =0.2$ . For (**a**) $\lambda _{1}=0.03$, $\nu =1.5$, $\lambda _{2}=0.3$. For (**b**) we used $\beta _3(D)$ as the transmission rate, $\lambda _{1}=0.03$ and $\lambda _2=0.3$. For (**c**) we also used $\beta _3(D)$ as the transmission rate, $\lambda _{1}=0.03$ and $\nu =1.5$. For (**d**) $\lambda _{2}=0.3$, $\nu =1.5$ and $\beta _3(D)$ as the transmission rate. The initial condition $D(0)=D_*$ was used for each type of society.

**Figure 8:**
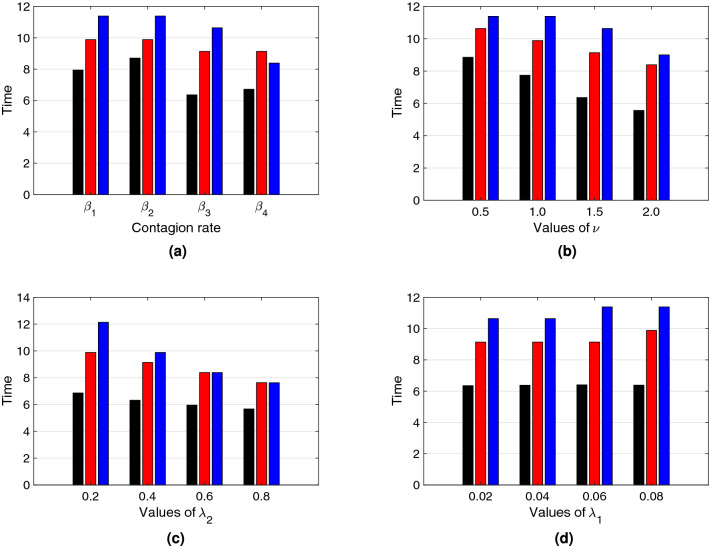
Time of occurrence of the peak of a first epidemic outbreak from the system in Eq. (1) with respect to, (**a**) contagion rates $\beta _i$, $i=1,2,3,4$, (**b**) measure of decrease of transmission rate with distance $\nu$, (**c**) reaction velocity to change $\lambda _2$, (**d**) rate of resistance to change $\lambda _1$, for different society types: Black, red and blue bars correspond to $D_*=0.75\;({\rm m})$, $D_*=1.05\;({\rm m})$, and $D_*=1.35\;({\rm m})$ respectively. These correspond to societies of small ([0, 100)), medium ([100, 120]) and large ($[120,\infty )$) *natural-distance* types respectively (see Table 1). The height of each bar represents the time of occurrence of the peak of the epidemic curve. The other parameter values were taken to be $\beta _{*}=0.5$ and $\gamma =0.2$. For (**a**) $\nu =1.5$, $\lambda _{2}=0.3$. For (**b**) we used $\beta _3(D)$ as the transmission rate, $\lambda _{1}=0.03$ and $\lambda _2=0.3$. For (**c**) we also used $\beta _3(D)$ as the transmission rate, $\lambda _{1}=0.03$ and $\nu =1.5$. For (**d**) $\lambda _{2}=0.3$, $\nu =1.5$ and $\beta _3(D)$ as the transmission rate. The initial condition $D(0)=D_*$ was used for each type of society.

Figures 7 and 8 depict bar plots that illustrate, respectively, the height of the peak of a first epidemic outbreak and its time of occurrence for: (a) each transmission rate $\beta _i(D)$, $i\in \{1,2,3,4\}$; (b) different $\nu$ values ($\nu \in \{0.5, 1.0, 1.5, 2.0]$); (c) different $\lambda _2$ values ($\lambda _2 \in \{0.2, 0.4, 0.6, 0.8\}$); (d) different $\lambda _1$ values ($\lambda _1 \in \{0.02, 0.04, 0.06, 0.08\}$); each for the three different types of social *natural-distance* (small, medium, large) represented by colors (black, red, blue). Figure 7a for instance, shows that $\beta _{3}(D)$ returns the highest peak compared to the other transmission rate functional forms, for small social *natural-distance* societies (black), and also that the difference in peak size between different societies is biggest for $\beta _3(D)$ and $\beta _4(D)$. Figure 7b shows that for societies of small *natural-distance* type, the higher the $\nu$ value, the higher is the epidemic peak, and that the contrary is true for societies of large *natural-distance* type. Figure 7c depicts that the higher the reaction velocity to change ($\lambda _{2}$) is, the lower is the infection peak, especially noticeable for societies of small *natural-distance* types, and Fig. 7d illustrates that for the parameter range chosen, there is little effect on peak size of the rate ($\lambda _1$) at which individuals return to their *natural-distance* $D_*$. In general, all four subplots show that the smaller the *natural-distance* type of a society is, the larger is the epidemic peak size.

Additionally, Fig. 8 shows in general that, the smaller the *natural-distance* type of a society is, the sooner occurs the peak. In particular, Fig. 8a depicts that for $\beta _3(D)$ and for societies of small *natural-distance* type, the peak occurs the earliest. On the other hand, in Fig. 8b we observe that, the larger the value for $\nu$ is, the sooner is the timing of the peak. Figure 8c depicts that the time of the peak does not experience such a great change according to $\lambda _2$ compared to the effect on peak size, especially for societies of small *natural-distance* type; but, for societies of large *natural-distance* type, increasing $\lambda _2$ may have an effect on earlier peak occurrence. So, comparing Fig. 7c with Fig. 8c, especially for societies of medium and large *natural-distance* type (red, blue), one can observe that, the larger $\lambda _2$, the smaller is the peak but, at the same time, the sooner it occurs. Hence, there is a trade off between reduced peak size and early occurrence of the peak. Finally, Fig. 8d illustrates that the greater $\lambda _1$, the later the peak may occur, mainly for societies of medium or large *natural-distance* types.

### Temporal dynamics of the transmission rates impacted by *interaction-distance* (*D*(*t*))

**Figure 9:**
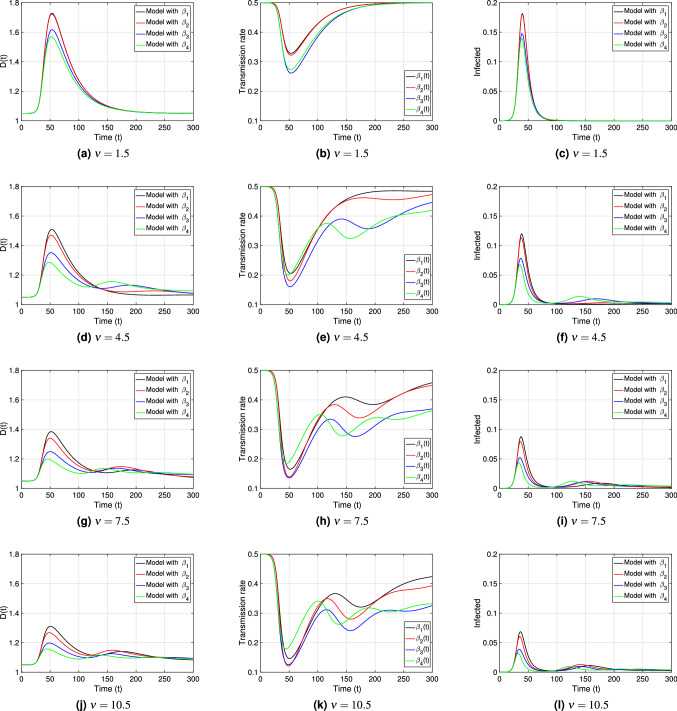
Temporal evolution of the *interaction-distance* *D*(*t*) (fist column); the transmission rates $\beta _i(t)=\beta _i(D(t))$, $i=1,2,3,4$, from Eqs. (6)–(9) (second column); and the point prevalence *I*(*t*)/*N* (third column); for $\nu =1.5, 4.5, 7.5, 10.5$. The remaining parameter values were taken to be $\beta _{*}=0.5$; $\gamma =0.2$, $\lambda _{1}=0.03$, $\lambda _{2}=0.3$ and $D_*=1.05=\bar{D}_*$, with ${\mathscr {R}}_0=2.5$. The initial conditions used are $S(0)=0.99999$, $I(0)=0.00001$, $R(0)=0$ and $D(0)=D_*$.

Figure 9 shows the evolution in time of the *interaction-distance* *D*(*t*), the four transmission rates from Eqs. (6)–(9), and the point prevalence *I*(*t*)/*N* for different $\nu$ values. Since larger $\nu$ values account for important differences among societies during the initial period of disease propagation (see Figs. 3, 4, and 5) we choose $\nu \in \{1.5, 4.5, 7.5, 10.5\}$ in the larger range. We describe in Fig. 9 the practical significance of the different transmission rate functional forms, their correlation with *interaction-distance*, and their impact on the curves of infected; in the setting of an average society with *natural-distance* $D_*=1.05$. We first describe general temporal features common to all four transmission rate functions and then point out specific characteristics that make them differ in their practical significance for disease modeling.

Upon the arrival of an infectious disease with high morbidity and/or mortality, a decrease of the transmission rate during the initial period of disease expansion can be observed^28, 48, 49^. We can observe in Fig. 9, that our model describes that behavior for the transmission rates $\beta _i(t)$, $i=1,2,3,4$. Additionally, one of the novelties of our model is that it explains the decreasing behavior of the transmission rates by a behavioral change in the population, represented by social distancing; i.e., it shows that the initial decrease in the transmission rates may be due to an increment of the *interaction-distance* *D*(*t*), whose dynamic depends on the increase in active cases and some correlated behavioral factor (see the equation for *D*(*t*) in Eq. (1)). In fact, just as we have observed previously in Fig. 6 for $\beta _3(\cdot )$, in Fig. 9 we see that for all four transmission rates, during the first 50 days of disease propagation, the *interaction-distance* *D*(*t*) increases (see Fig. 9a,d,g,j), which produces a reduction in the transmission rate during the same time period (see Fig. 9b,e,h,k) and a first epidemic peak in that time frame (see Fig. 9c,f,i,l).

During the course of a pandemic, the change in social distancing behavior affects the rate of efficient contacts for disease transmission and, therefore, the transmission rate. The rising or falling of the transmission rate is one of the reasons that explains the change in the effective reproduction number—a dynamic measure of the average number of secondary cases per infected case in a population composted by susceptible and non-susceptible individuals—that has been observed during epidemic outbreaks, since this measure is a function of the efficient contacts, among others^50–55^. Our model, with its different transmission rate functions correlated to distancing behavior, gives a range of practical scenarios for the evolution of a changing transmission rate responsible for disease propagation. This evolution is characterized by the transmission rate functions given in Eqs. (6)–(9) that are defined by $\nu$ and their dependency on *D*(*t*).

First, we observe how the parameter $\nu$ affects the characterization of disease transmission in general. We see from Fig. 9 that after the first minimum value of each transmission rate, the rates start increasing, tending to return to their initial state $\beta _*$. We observe that the convergence to their initial value happens in a shorter time-frame for small $\nu$ values than for large ones. Indeed, we can see clearly from Fig. 9b,e,h,k, that for instance $\forall t>100$, $\beta _{i}(D,\nu _{1})(t)>\beta _{i}(D,\nu _{2})(t)$, for $\nu _{1}<\nu _{2}$, $\forall i=1,2,3,4$ and $D> D_{*}$. We also observe that oscillations appear for larger $\nu$ values during the recovery phase of the transmission rates, which we will discuss in more detail below.

It is important to add to the discussion how the dependency on the *interaction-distance* *D*(*t*) of the different transmission rates affect their timely evolution. Observe that the efficiency of *D* in lowering each transmission rate differs for different $\nu$ values: the larger $\nu$ is, the more efficient is an absolute increase/reduction in *interaction-distance* in reducing/increasing each transmission rate; e.g., only a small increment in *D*(*t*) from $t=0$ to $t=50$ is necessary to achieve a significant reduction in each transmission rate during that time period. As a consequence, observe that for large $\nu$ values, only a small initial increase in the *interaction-distance* produces a low first epidemic peak. That efficiency of *D* in reducing each transmission rate is at a cost: a low first epidemic peak in exchange for breaking the unimodality of the active-infected-curve produced by the classical SIR model with constant transmission rate (one bell-shaped infection curve due to the epidemic growth being limited by the proportion of susceptible individuals)^63, 64^, and hence our model may produce several further epidemic peaks (see Fig. 9c,f,i,l). This is a direct consequence of the oscillatory recovery of the transmission rate mentioned before, produced by the oscillatory behavior of *D*(*t*) in combination with its efficiency in reducing transmission.

Next, we will discuss some specific characteristics of the transmission rate functions. We observe from Fig. 9e,h,k differences between the four transmission rates in their oscillations that describe disease dynamics: oscillations of $\beta _4(\cdot )$(in green) for any $\nu$ value are ahead of the oscillations of any of the other three transmission rates, producing earlier epidemic peaks; on the contrary, $\beta _1(\cdot )$ (in black) produces oscillations the latest, producing later epidemic peaks; the transmission rate function pairs $\beta _1$ and $\beta _2$, and $\beta _3$ and $\beta _4$ generate similar dynamic behavior for small $\nu$ values, but their behavior drifts apart for increasing $\nu$. Also, we can see in Fig. 9f,i,l that peak sizes and time-spans between peaks change according to different transmission rate functions and their oscillatory shape.

## Discussion and conclusions

To control the spread of a disease causing an epidemic or pandemic, the only effective measure may be to reduce the effective contact rate by social distancing. In fact, there is scientific evidence that suggests that the transmission of pathogenic agents occurs with sensitivity to human behavior, in particular to the distance between individuals^24, 65^. The importance of social distancing—to keep infectious diseases from spreading and mitigate their morbidity and mortality—was revealed in a historic article that studied the data of Pneumonic Plague in Manchurian in north-eastern Asia during the years 1910-11 and 1920-21^65^. That study evidenced an epidemiological risk for pneumonia for distances between 5 *cm* to 2 *m*. This makes it clear that incorporating into mathematical models the factor of social distance is important if more precision is needed to sustain and guide measures of sanitary intervention^24^. Our simple model supports these findings.

The model results describe how the distance that individuals keep from each other varies in time and with respect to point prevalence (see Fig. 6). In particular, the simulations illustrate that the first peak in distancing after the onset of an epidemic (the moment when people keep the largest distance from each other) occurs—as a reactive reaction—after the first peak of infections happens, varying the time of occurrence according to the type of society. We could also observe that societies where people keep a small *natural-distance* from each other, have to change their distancing behavior the most to counteract disease spread (see Fig. 6b).

Our results in Figs. 7 and 8 confirm the importance of social distancing, and show differences in peak size and peak time of a first epidemic outbreak for different cultural settings. In particular, our results show clearly the vulnerability of societies of small social *natural-distance* type—in which individuals maintain a distance of less than one meter from each other. Such societies could experience a mayor epidemic peak that occurs early after the onset of the epidemic. On the other hand, societies in which individuals maintain a distance from each other of more than one meter, experience a lower peak that occurs later after the beginning of the epidemic, as compared to peak size and time for other types of societies.

Our simulations also show differences in peak size and time for different epidemiological and social distancing related parameters for each society type, during a first epidemic outbreak. For instance, the form of the transmission rate—which is distance dependent—affects greatly size and time of the epidemic peak. Also, parameters related to how fast individuals change behavior according to point prevalence levels ($\lambda _2$) and how resistant ($\lambda _1$) individuals are to change their natural distance ($D_*$), may be key for disease dynamics. For instance, in general, populations that react quickly to the observed point prevalence experience smaller peaks, which is specially pronounced for societies of small *natural-distance* type (see Fig. 7c); but, for small peak sizes, there is a trade off: and the peak may occur sooner, especially for societies of large *natural-distance* type (see Fig. 8c). Hence, a society of large *natural-distance* type that reacts fast to change when there is disease present, may experience a small but early first epidemic peak.

The shape of the infected curve beyond the first epidemic outbreak in a pandemic situation changes from country to country, as has been observed for instance during the current COVID-19 pandemic^66^. In particular, how close or how high possible further epidemic peaks are varies. Our numerical results show that the transmission rate functional forms used—since they are able to produce oscillations (see Fig. 9)—give us a range of possibilities that may help to describe the qualitative behavior of different infection curve scenarios. Additionally, we can explain a possible cause for the changing transmission rates in terms of a tangible variable: *interaction-distance* *D*(*t*); that describes the distancing behavior of individuals in time. We can also describe how efficient social distancing is in changing disease transmission (using the parameter $\nu$), which may vary for different populations. This efficiency determines the form of the oscillatory behavior of the transmission rates and hence, the appearance of several further peaks; this way breaking the unimodality of the active-infected-curve produced by the classical SIR model.

In order to obtain better guidelines from the model, we plan in future work to extend the model including more epidemiological classes and an additional structure that further describes human behavior in an epidemic situation. For instance, for modeling COVID-19, additional classes for pre-symptomatic, asymptomatic and hospitalized individuals may be necessary. We also would like to conduct some sensitivity analysis. For instance, to compute the Partial Rank Correlation Coefficient (PRCC) for each parameter and parameter ranges could give insight into which parameters affect epidemic peak and peak time the most. We also would like to address, which of the four transmission rate functional forms would best fit for instance the COVID-19 epidemic data for different types of societies, as well as consider age-group differences, among other factors.

Our model and its results are a first approach for analyzing the effect of initiatives for pandemic preparedness under different epidemiological and cultural settings, determined by: (1) the transmission rate of a particular disease, which is inversely proportional to distance; (2) the velocity of the population to react to the presence of the disease ($\lambda _2$); (3) the resistance that individuals experience to change their natural distance ($\lambda _1$). Indeed, if the goal would be to reduce peak size during a first epidemic outbreak and postpone its timing (for instance to gain time to implement proper healthcare conditions to treat infected individuals) and the society affected is of small social *natural-distance* type (less than one meter), then, measures that change the society type—by increasing the natural distance given by the culture ($D_*$) to more than one meter—would lower the peak and postpone it. Such measures in the short term could be for instance quarantine, and in the long term cultural re-education initiatives that change the distancing behavior of the population. Changing the *natural-distance* that people keep from each other- in other words, to change society type- may be more effective for lowering the peak of a first epidemic outbreak than not changing society type and instead, finding measures that increase public health awareness and improve the velocity of reaction to change ($\lambda _2$) of the society. Indeed, for instance for societies of large *natural-distance* type, to increase the reaction velocity may anticipate the peak, which may not be desired.

Control measures such as quarantine, indeed aim to stop social activities as a way to obtain large social distancing, and this way increase the *natural-distance* given by the culture. Without those measures, it is extremely difficult to control that our personal space is respected by others, especially in cultures of small *natural-distance* type. Government imposed control measures are not sustainable in the long term, and hence cultural re-education initiatives are necessary to get individuals accustomed to change their social behavior. A cultural change is necessary. As our results show, in general, societies that show during a first outbreak the smallest peak size, occurring late after the onset of the epidemics, are societies where the *natural-distance* given culturally is large (individuals upon encounter maintain a distance larger than 1.2 meters from each other), almost independent of the transmission rate form (see Figs. 7a,b and 8a,b).

Even though it is not easy to change habits acquired throughout the years, it is our obligation to make the change. We have to insist that public health authorities and their technical advisors, as well as individuals in the population, impulse initiatives for cultural re-education to confront epidemics to come. As stated in^16^, while learning from history, now may be our opportunity to make progress in this direction.
